# Chicago Medical Society, August 17, 1874

**Published:** 1874-10-01

**Authors:** 

**Affiliations:** Reporter Pro Tem.


### CHICAGO MEDICAL SOCIETY.

### Meeting of August 17th.

*Dr. Graham, Reporter Pro Tem.*

THE President, Dr. Quine in the chair. Dr. Stillians presented a specimen of intestine, showing intussusception of the colon at five different points, three of which were well marked. The patient was a boy five years old. The only symptoms at first were constipation and pretty severe colicky pains, for which anodyne and cathartic enemata were given. There was afterward considerable flatulence, and a tender point was discovered below and to the right of the umbilicus. A very small quantity of fæces was found, at two or three different times, after giving the injections. During the second day the skin became hot and the pulse rapid and feeble, which symptoms continued and became more prominent—death ensuing that night.

In answer to a question the Dr. stated that there had not been any vomiting, or discharge of blood from the bowels.

Dr. Dyas said it was very unusual not to have these symptoms, and that there was generally a tumor on right side. Death was often caused by the shock to the system, without any symptoms of inflammation being present. Purgatives should not be given in such cases, but enemata of simple water and opiates should be chiefly relied on.

D. A. H. Foster next read the report of a case of hydrophobia.

On motion the discussion of this paper was deferred till the next meeting.

Dr. H. M. Lyman read the report of his observations on three cases of *Diabetes Mellitus* occurring in the County Hospital during his term of service:

*Diabetes Mellitus.* Three cases of this disease were under treatment. One died; another left the hospital after remaining three months under treatment; the third was still in the house at the close of my term of service. He was a man in middle life, whose case presented no unusual symptoms. The boy who left us was of Swedish parentage, aet. 17 years; admitted to the hospital April 18th, 1874. Had never noticed any failure of health till about March 1st, when he began to experience great thirst, dryness of the skin, frequent and copious urination, dull aching in the legs, progressive weakness, a ravenous appetite, and some indistinctness of vision. On admission, his pulse, temperature and respiration were normal, but the skin was dry and desquamating, and his tongue was red and dry in the centre. During the first twenty-four hours after admission the patient voided twelve pints of urine,

(sp. gr. 1032) which gave a decidedly saccharine reaction, with Sehling's test. He was ordered the most nitrogenous diet which our somewhat limited resources could supply; was douched with cold water every morning, and took, once every four hours, a scruple of bromide of potassium, with an equal quantity of chlorate of potassium. He had ten grains of Dover's powder every night.

April 24th. Voided seventeen pints and a half.

This quantity gradually diminished till May 3d, when it was only nine pints. At this point it remained stationary until May 12th, when the treatment was changed in order to make trial of a recent German theory of the disease, and the patient took nothing but five grains of carbolic acid three times daily. This treatment proved unsuccessful, for the quantity of urine rapidly increased. May 22d it was fourteen pints; June 1st, sixteen pints; June 16th, eighteen pints. Medicine now seemed to exercise very little control over the disease; but when the uncommonly hot weather in July began to stimulate the functions of the skin, the urinary discharge was rapidly diminished; and when the patient left the hospital, July 18th, he voided only six pints *per diem*. In every other particular, however, his condition was unchanged.

The diabetic patient who died under treatment was an Irish laborer, aet. 22 years; admitted to the hospital Feb. 11th, 1874. For about six months he had been losing flesh and strength. Had experienced a slight tracheal irritation, but did not actually begin to cough till three weeks before entering the hospital. On admission he was very much emaciated, his face was flushed, skin harsh and dry, tongue clean, appetite good, bowels costive, urine copious (sp. gr. 1020) and saccharine. A slight cough attracted attention to his lungs,—respiration was largely abdominal, and the respiratory sounds were exaggerated. He was ordered to take cod-liver oil, with citrate of iron and quinine. The quantity of urine gradually diminished from fifteen pints per diem till May 5th, when it was only eight pints. He was now ordered to take carbolic acid instead of iron and quinine, but no benefit was derived from the change. As the weather grew warm he voided less water; but his disease progressed, and he died July 14th. *Autopsy* thirty hours after death. Extreme dryness of all the tissues was remarked. The brain was very anæmic, but otherwise healthy. The condition of the left lung was normal, but the lower lobe of the right lung was tubercular and contained several suppurating cavities. The liver and spleen were of the usual weight, but were filled with masses of tubercle, which had in several places degenerated into tubercular abscesses. A similar abscess, as large as a pea, was found upon the greater convexity of the left kidney. The other viscera presented no unusual appearance.

Dr. J. S. Knox gave the details of a case of uterine disease, in which there was sloughing of the entire cervix uteri. The patient had been under treatment for twenty-seven years, by many different physicians. Had submitted to leechings, scarifications, cautery, fomentations, douches, paintings with iodine and chromic acid, and had tried "pessaries enough to support the womb of time through all eternity." She came under the

Dr.'s care by applying for re-adjustment of a Hodge pessary, which she had worn for a long time, which was removed on account of the inflammation to which it seemed to have given rise, and emollient treatment was pursued several weeks, when the sloughing of the cervix took place. The rapid healing of the stump, and the absence of every sign or symptom which would cause a suspicion of malignant disease, led the Dr. to conclude that the sloughing was the result of diminished vascularity and vitality, due to the many recurring inflammations, and the abundant treatment of previous years.

Dr. W. E. Clark exhibited the sack of an ovarian tumor, which weighed fifty pounds, which he had removed that afternoon—both ovaries were diseased, and there was also a fibroid tumor of the uterus.

A motion was made and passed, after considerable discussion, to appoint a committee to co-operate with similar committees from the Society of Physicians and Surgeons, and the College of Pharmacy, in considering the relations of physicians and pharmacists, and to consider and report any measures thought necessary to check the prescribing of proprietary medicines. Drs. C. W. Earle, A. H. Foster, and S. C. Stillians, were appointed such committee.

On motion society adjourned.

